# Supplementary material for: Modelling Skylarks (Alauda arvensis) to Predict Impacts of Changes in Land Management and Policy: Development and Testing of an Agent-Based Model
Source: PLoS One. 2013 Jun 6;8(6):e65803. doi: 10.1371/journal.pone.0065803 (PMC3675089; doi:10.1371/journal.pone.0065803)
Supplement: Supporting Information S4 — The skylark ODdox as a zipped archive. (ZIP) [file pone.0065803.s004.zip › Skylark_ODdox/globals_0x63.html]

ALMaSS Skylark ODdox: File Members


ALMaSS Skylark ODdox
 2.0


- Main Page
- Related Pages
- Classes
- Files

- File List
- File Members

- All
- Functions
- Variables
- Typedefs
- Enumerations
- Enumerator
- Macros

- \_
- a
- b
- c
- d
- e
- f
- g
- h
- i
- j
- k
- l
- m
- n
- o
- p
- r
- s
- t
- u
- v
- w
- x

Here is a list of all file members with links to the files they belong to:

### - c -

- c\_insolation
  : weather.h
- c\_SolarConversion
  : elements.cpp
- cattle\_out
  : treatment.h
- cattle\_out\_low
  : treatment.h
- CE\_nest
  : skylarks\_all.cpp
- cfg\_AddBeetleBanks
  : Landscape.cpp
- cfg\_adultreturnmort
  : skylarks\_all.cpp
  , SkylarkConfigs.cpp
- cfg\_BeetleBankChance
  : Landscape.cpp
- cfg\_beetlebankinsectscaler
  : elements.cpp
- cfg\_BeetleBankMaxArea
  : Landscape.cpp
- cfg\_BeetleBankWidth
  : Landscape.cpp
- CFG\_BOOL
  : configurator.h
- cfg\_Breed\_Res\_Thresh1
  : SkylarkConfigs.cpp
  , skylarks\_all.cpp
- cfg\_CalculateCentroids
  : Landscape.cpp
- cfg\_CatastropheEventStartYear
  : PopulationManager.cpp
- CFG\_CHANNEL\_BITS
  : pesticide.cpp
- CFG\_CHANNEL\_MAXVAL
  : pesticide.cpp
- cfg\_CIPEGridOutput\_day
  : PopulationManager.cpp
- cfg\_CIPEGridOutput\_day\_b
  : PopulationManager.cpp
- cfg\_CIPEGridOutput\_filename
  : PopulationManager.cpp
- cfg\_CIPEGridOutput\_filenameB
  : PopulationManager.cpp
- cfg\_CIPEGridOutput\_Interval
  : PopulationManager.cpp
- cfg\_CIPEGridOutput\_used
  : PopulationManager.cpp
  , skylarks\_all.cpp
- cfg\_CipeGridSize
  : PopulationManager.cpp
- cfg\_ClutchMortProb
  : SkylarkConfigs.cpp
  , skylarks\_all.cpp
- cfg\_ConversionEffReduc
  : SkylarkConfigs.cpp
  , skylarks\_all.cpp
- cfg\_Cooling\_Rate\_Eggs
  : SkylarkConfigs.cpp
  , skylarks\_all.cpp
- CFG\_CUSTOM
  : configurator.h
- cfg\_DayInMonth
  : ALMaSS\_CmdLine.cpp
  , PopulationManager.cpp
- cfg\_densityconstant\_a
  : SkylarkConfigs.cpp
  , skylarks\_all.cpp
- cfg\_densityconstant\_b
  : SkylarkConfigs.cpp
  , skylarks\_all.cpp
- cfg\_densityconstant\_c
  : SkylarkConfigs.cpp
  , skylarks\_all.cpp
- cfg\_dumpvegjan
  : Landscape.cpp
  , misc.cpp
  , ALMaSS\_CmdLine.cpp
- cfg\_dumpvegjanfile
  : Landscape.cpp
  , misc.cpp
  , ALMaSS\_CmdLine.cpp
- cfg\_dumpvegjune
  : Landscape.cpp
  , misc.cpp
  , ALMaSS\_CmdLine.cpp
- cfg\_dumpvegjunefile
  : Landscape.cpp
  , misc.cpp
  , ALMaSS\_CmdLine.cpp
- cfg\_EggTemp
  : SkylarkConfigs.cpp
  , skylarks\_all.cpp
- cfg\_ele\_weedscaling
  : elements.cpp
- cfg\_EM\_Nestling\_a
  : SkylarkConfigs.cpp
  , skylarks\_all.cpp
- cfg\_EM\_Nestling\_b
  : SkylarkConfigs.cpp
  , skylarks\_all.cpp
- cfg\_fecundity\_reduc
  : SkylarkConfigs.cpp
  , skylarks\_all.cpp
- cfg\_fecundity\_reduc\_chance
  : SkylarkConfigs.cpp
  , skylarks\_all.cpp
- cfg\_FemaleMinTerritoryAcceptScore
  : SkylarkConfigs.cpp
  , skylarks\_all.cpp
- cfg\_fixed\_random\_sequence
  : ALMaSS\_CmdLine.cpp
  , PopulationManager.cpp
- CFG\_FLOAT
  : configurator.h
- cfg\_FoodTripsPerDay
  : SkylarkConfigs.cpp
  , skylarks\_all.cpp
- cfg\_fungi\_app\_prop
  : Landscape.cpp
- cfg\_greg\_app\_prop
  : Landscape.cpp
- cfg\_HedgeSubtypeMaximum
  : Landscape.cpp
- cfg\_HedgeSubtypeMinimum
  : Landscape.cpp
- cfg\_heightconstant\_a
  : SkylarkConfigs.cpp
  , skylarks\_all.cpp
- cfg\_heightconstant\_b
  : SkylarkConfigs.cpp
  , skylarks\_all.cpp
- cfg\_heightconstant\_c
  : SkylarkConfigs.cpp
  , skylarks\_all.cpp
- cfg\_herbi\_app\_prop
  : Landscape.cpp
- cfg\_hindconstantD\_b
  : SkylarkConfigs.cpp
  , skylarks\_all.cpp
- cfg\_hindconstantH\_b
  : SkylarkConfigs.cpp
  , skylarks\_all.cpp
- cfg\_HQualityBareEarth
  : SkylarkConfigs.cpp
  , skylarks\_all.cpp
- cfg\_HQualityHedge
  : SkylarkConfigs.cpp
  , skylarks\_all.cpp
- cfg\_HQualityHedgeScrub
  : SkylarkConfigs.cpp
  , skylarks\_all.cpp
- cfg\_HQualityMetalRoad
  : SkylarkConfigs.cpp
  , skylarks\_all.cpp
- cfg\_HQualityNeutral
  : SkylarkConfigs.cpp
  , skylarks\_all.cpp
- cfg\_HQualityOpenTallVeg
  : SkylarkConfigs.cpp
  , skylarks\_all.cpp
- cfg\_HQualityTall
  : SkylarkConfigs.cpp
  , skylarks\_all.cpp
- cfg\_HQualityTall2
  : SkylarkConfigs.cpp
  , skylarks\_all.cpp
- cfg\_HQualityTallVeg
  : SkylarkConfigs.cpp
  , skylarks\_all.cpp
- cfg\_HQualityTrack
  : SkylarkConfigs.cpp
  , skylarks\_all.cpp
- cfg\_HQualityVeg30cm
  : SkylarkConfigs.cpp
  , skylarks\_all.cpp
- cfg\_HQualityWater
  : SkylarkConfigs.cpp
  , skylarks\_all.cpp
- cfg\_ins\_app\_prop
  : Landscape.cpp
- cfg\_insecticide\_direct\_mortE
  : SkylarkConfigs.cpp
  , skylarks\_all.cpp
- cfg\_insecticide\_direct\_mortF
  : SkylarkConfigs.cpp
  , skylarks\_all.cpp
- cfg\_insecticide\_direct\_mortM
  : SkylarkConfigs.cpp
  , skylarks\_all.cpp
- cfg\_insecticide\_direct\_mortN
  : SkylarkConfigs.cpp
  , skylarks\_all.cpp
- cfg\_insecticide\_direct\_mortP
  : SkylarkConfigs.cpp
  , skylarks\_all.cpp
- CFG\_INT
  : configurator.h
- cfg\_juvreturnmort
  : SkylarkConfigs.cpp
  , skylarks\_all.cpp
- cfg\_l\_treatment\_size
  : Landscape.cpp
- cfg\_l\_treatment\_x
  : Landscape.cpp
- cfg\_l\_treatment\_y
  : Landscape.cpp
- cfg\_l\_usecustompoly
  : Landscape.cpp
- cfg\_MaleSplitScale
  : SkylarkConfigs.cpp
  , skylarks\_all.cpp
- cfg\_manipulation1\_on
  : rastermap.cpp
- cfg\_map\_usesoiltypes
  : Landscape.cpp
- CFG\_MAX\_LINE\_LENGTH
  : configurator.h
- cfg\_maxfeedrain
  : SkylarkConfigs.cpp
  , skylarks\_all.cpp
- cfg\_MD\_Threshold
  : SkylarkConfigs.cpp
  , skylarks\_all.cpp
- cfg\_MeanExtractionRatePerMinute
  : SkylarkConfigs.cpp
  , skylarks\_all.cpp
- cfg\_MeanHatchingWeight
  : SkylarkConfigs.cpp
  , skylarks\_all.cpp
- cfg\_MinDaysToHatch
  : SkylarkConfigs.cpp
  , skylarks\_all.cpp
- cfg\_mintownbuildingdistance
  : Landscape.cpp
- cfg\_mintownbuildingnumber
  : Landscape.cpp
- cfg\_NestLeavingChance
  : SkylarkConfigs.cpp
  , skylarks\_all.cpp
- cfg\_NestLeavingWeight
  : SkylarkConfigs.cpp
  , skylarks\_all.cpp
- cfg\_NestlingMortProb
  : SkylarkConfigs.cpp
  , skylarks\_all.cpp
- cfg\_NestPlacementMinQual
  : SkylarkConfigs.cpp
  , skylarks\_all.cpp
- CFG\_NONE
  : configurator.h
- cfg\_OrchardNoCutsDay
  : elements.cpp
- cfg\_OrchardSprayDay
  : elements.cpp
- cfg\_OrchardSprayDay2
  : elements.cpp
- cfg\_organic\_extensive
  : farm.cpp
- cfg\_PatchyPremium
  : SkylarkConfigs.cpp
  , skylarks\_all.cpp
- cfg\_PEmax
  : SkylarkConfigs.cpp
  , skylarks\_all.cpp
- cfg\_pest\_productapplic\_enddate
  : pesticide.cpp
- cfg\_pest\_productapplic\_period
  : elements.cpp
  , pesticide.cpp
- cfg\_pest\_productapplic\_startdate
  : pesticide.cpp
  , elements.cpp
- cfg\_pest\_productapplic\_startdate2
  : elements.cpp
  , pesticide.cpp
- cfg\_pest\_residue\_or\_rate
  : pesticide.cpp
- cfg\_pesticidetesttype
  : Landscape.cpp
- cfg\_pm\_eventfrequency
  : PopulationManager.cpp
  , skylarks\_all.cpp
- cfg\_pm\_eventsize
  : skylarks\_all.cpp
  , PopulationManager.cpp
- cfg\_PreFledgeMortProb
  : SkylarkConfigs.cpp
  , skylarks\_all.cpp
- CFG\_PRIVATE
  : configurator.h
- cfg\_productapplicendyear
  : Landscape.cpp
- cfg\_productapplicstartyear
  : Landscape.cpp
- CFG\_PUBLIC
  : configurator.h
- cfg\_rainhindpow
  : skylarks\_all.cpp
  , SkylarkConfigs.cpp
- cfg\_rastermapmanipmultiply
  : rastermap.cpp
- cfg\_ReallyBigOutput\_day1
  : PopulationManager.cpp
- cfg\_ReallyBigOutput\_day2
  : PopulationManager.cpp
- cfg\_ReallyBigOutput\_day3
  : PopulationManager.cpp
- cfg\_ReallyBigOutput\_day4
  : PopulationManager.cpp
- cfg\_ReallyBigOutput\_filename
  : PopulationManager.cpp
- cfg\_ReallyBigOutput\_interval
  : PopulationManager.cpp
- cfg\_ReallyBigOutput\_used
  : PopulationManager.cpp
  , skylarks\_all.cpp
- cfg\_ReallyBigOutputFirstYear
  : PopulationManager.cpp
- cfg\_ReallyBigOutputMonthly\_used
  : skylarks\_all.cpp
- cfg\_RipleysOutput\_day
  : PopulationManager.cpp
- cfg\_RipleysOutput\_filename
  : PopulationManager.cpp
- cfg\_RipleysOutput\_interval
  : PopulationManager.cpp
- cfg\_RipleysOutput\_used
  : skylarks\_all.cpp
  , PopulationManager.cpp
- cfg\_RipleysOutputFirstYear
  : PopulationManager.cpp
- cfg\_RipleysOutputMonthly\_used
  : PopulationManager.cpp
- cfg\_rodenticide\_BLcountry\_AnnFreq
  : Rodenticide.cpp
- cfg\_rodenticide\_BLcountry\_Length
  : Rodenticide.cpp
- cfg\_rodenticide\_BLtown\_AnnFreq
  : Rodenticide.cpp
- cfg\_rodenticide\_BLtown\_Length
  : Rodenticide.cpp
- cfg\_rodenticide\_BLwoodland\_AnnFreq
  : Rodenticide.cpp
- cfg\_rodenticide\_BLwoodland\_Length
  : Rodenticide.cpp
- cfg\_rodenticide\_deathRate
  : Rodenticide.cpp
- cfg\_rodenticide\_diffusionRate
  : Rodenticide.cpp
- cfg\_rodenticide\_dumpmapday
  : Rodenticide.cpp
- cfg\_rodenticide\_dumpmapendyear
  : Rodenticide.cpp
- cfg\_rodenticide\_dumpmapstartyear
  : Rodenticide.cpp
- cfg\_rodenticide\_dumppolyendyear
  : Rodenticide.cpp
- cfg\_rodenticide\_dumppolyinterval
  : Rodenticide.cpp
- cfg\_rodenticide\_dumppolystartyear
  : Rodenticide.cpp
- cfg\_rodenticide\_enable
  : Landscape.cpp
- cfg\_rodenticide\_gridsize
  : Rodenticide.cpp
- cfg\_rodenticide\_immigrationRate
  : Rodenticide.cpp
- cfg\_rodenticide\_reporting\_enable
  : Landscape.cpp
- cfg\_silage\_prop
  : Landscape.cpp
- cfg\_SK\_hindrance\_scale
  : skylarks\_all.cpp
- cfg\_sk\_triplength
  : skylarks\_all.cpp
  , SkylarkConfigs.cpp
- cfg\_SkScrapesPremiumII
  : SkylarkConfigs.cpp
  , skylarks\_all.cpp
- cfg\_SkStartNos
  : skylarks\_all.cpp
  , SkylarkConfigs.cpp
- cfg\_SkTramlinesPremium
  : skylarks\_all.cpp
  , SkylarkConfigs.cpp
- cfg\_Skylark\_female\_Biodegredation
  : SkylarkConfigs.cpp
  , skylarks\_all.cpp
- cfg\_Skylark\_female\_NOEL
  : skylarks\_all.cpp
  , SkylarkConfigs.cpp
- cfg\_Skylark\_male\_Biodegredation
  : SkylarkConfigs.cpp
  , skylarks\_all.cpp
- cfg\_Skylark\_male\_NOEL
  : skylarks\_all.cpp
  , SkylarkConfigs.cpp
- cfg\_Skylark\_nestling\_Biodegredation
  : SkylarkConfigs.cpp
  , skylarks\_all.cpp
- cfg\_Skylark\_nestling\_NOEL
  : skylarks\_all.cpp
  , SkylarkConfigs.cpp
- cfg\_Skylark\_prefledegling\_Biodegredation
  : skylarks\_all.cpp
  , SkylarkConfigs.cpp
- cfg\_Skylark\_prefledegling\_NOEL
  : skylarks\_all.cpp
  , SkylarkConfigs.cpp
- cfg\_strigling\_clutch
  : SkylarkConfigs.cpp
  , skylarks\_all.cpp
- cfg\_strigling\_nestling
  : skylarks\_all.cpp
  , SkylarkConfigs.cpp
- cfg\_strigling\_preflg
  : skylarks\_all.cpp
  , SkylarkConfigs.cpp
- cfg\_strigling\_prop
  : Landscape.cpp
- CFG\_STRING
  : configurator.h
- cfg\_temphindpow
  : skylarks\_all.cpp
  , SkylarkConfigs.cpp
- cfg\_tramline\_foraging
  : skylarks\_all.cpp
  , SkylarkConfigs.cpp
- cfg\_UMPatchyChance
  : elements.cpp
- cfg\_UseEasyPop
  : ALMaSS\_CmdLine.cpp
- cfg\_VoleCatastrophe\_interval
  : PopulationManager.cpp
  , ALMaSS\_CmdLine.cpp
- cfg\_VoleCatastrophe\_mortality
  : PopulationManager.cpp
  , ALMaSS\_CmdLine.cpp
- cfg\_VoleCatastrophe\_on
  : PopulationManager.cpp
  , ALMaSS\_CmdLine.cpp
- CfgSecureLevel
  : configurator.h
- CfgSecureStrings
  : configurator.cpp
- CfgType
  : configurator.h
- CfgTypeStrings
  : configurator.cpp
- CloseDownSim()
  : ALMaSS\_CmdLine.cpp
- ClutchMortProb
  : skylarks\_all.cpp
- CompareDist()
  : skylarks\_all.cpp
- CompPcts()
  : farm.cpp
- Cooling\_Rate\_Eggs
  : skylarks\_all.cpp
- CreateLandscape()
  : ALMaSS\_CmdLine.cpp
- CreatePopulationManager()
  : ALMaSS\_CmdLine.cpp
- cut\_to\_hay
  : treatment.h
- cut\_to\_silage
  : treatment.h
- cut\_weeds
  : treatment.h


- Generated on Thu Jan 10 2013 13:15:39 for ALMaSS Skylark ODdox by
   1.8.1.1
